# Efficacy of chest X-ray in the diagnosis of COVID-19 pneumonia: comparison with computed tomography through a simplified scoring system designed for triage

**DOI:** 10.1186/s43055-021-00541-x

**Published:** 2021-07-06

**Authors:** Akın Çinkooğlu, Selen Bayraktaroğlu, Naim Ceylan, Recep Savaş

**Affiliations:** Department of Radiology, Ege University Faculty of Medicine, 35100, Bornova, Izmir, Turkey

**Keywords:** Covid-19, Tomography, X-rays, Triage

## Abstract

**Background:**

There is no consensus on the imaging modality to be used in the diagnosis and management of Coronavirus disease 2019 (COVID-19) pneumonia. The purpose of this study was to make a comparison between computed tomography (CT) and chest X-ray (CXR) through a scoring system that can be beneficial to the clinicians in making the triage of patients diagnosed with COVID-19 pneumonia at their initial presentation to the hospital.

**Results:**

Patients with a negative CXR (30.1%) had significantly lower computed tomography score (CTS) (*p* < 0.001). Among the lung zones where the only infiltration pattern was ground glass opacity (GGO) on CT images, the ratio of abnormality seen on CXRs was 21.6%. The cut-off value of X-ray score (XRS) to distinguish the patients who needed intensive care at follow-up (*n* = 12) was 6 (AUC = 0.933, 95% CI = 0.886–0.979, 100% sensitivity, 81% specificity).

**Conclusions:**

Computed tomography is more effective in the diagnosis of COVID-19 pneumonia at the initial presentation due to the ease detection of GGOs. However, a baseline CXR taken after admission to the hospital can be valuable in predicting patients to be monitored in the intensive care units.

## Background

Coronavirus disease 2019 (COVID-19) is an infectious disease caused by the novel coronavirus ‘severe acute respiratory syndrome coronavirus-2’ [1]. Since December 2019, it has spread from China to worldwide and the World Health Organization declared a global pandemic on March 11, 2020 [2]. The infection can result in severe pneumonia and even fatal respiratory diseases such as acute respiratory distress syndrome [3]. Reverse transcription-polymerase chain reaction (RT-PCR) assay is the reference standard for definitive diagnosis. However, false-negative results may be seen at initial tests and cause delay in diagnosis [4]. Given the importance of early diagnosis and isolation of the patients, radiologic imaging modalities may be required, especially in patients with symptoms suggestive of pneumonia. Recent studies have demonstrated the high sensitivity of computed tomography (CT) [5, 6]. Bilateral, multifocal ground glass opacities (GGO), predominantly distributed in the lower and peripheral lung, have been reported as the most common CT features. Crazy paving pattern and consolidation have been described as progressive stage findings [7–10]. Although chest X-ray (CXR) findings mirror the findings described for CT, the sensitivity of CXR has been reported to be lower than those of CT and RT-PCR [11, 12]. However, CXR has several advantages over CT such as less ionizing radiation, rapid data acquisition, availability in the intensive care units (ICU), and portability [13]. There is still no consensus on the integrated use of chest CT and CXR in the management of COVID-19 pneumonia [14].

The purpose of this study was to make a comparison between chest CT and CXR using a scoring system that can also be beneficial to the clinicians in making the triage of patients diagnosed with COVID-19 pneumonia at their initial presentation to the hospital.

## Methods

This study was approved by the medical ethics committee of our institution (Approval Number = 20-12T/28). Informed consent for this retrospective study was waived.

### Patient selection

**Fig. 1:**
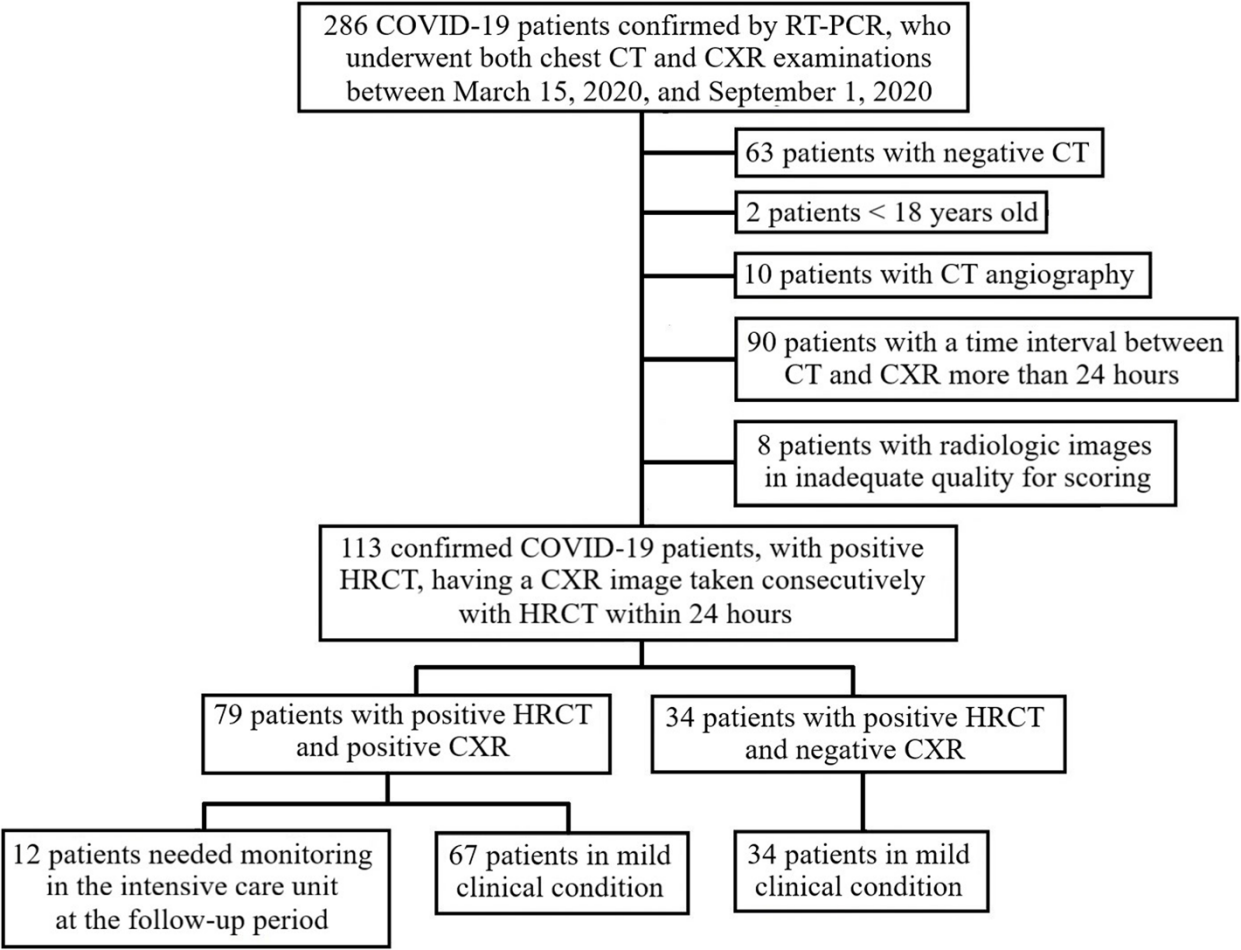
Flowchart of the patient selection. *CXR* chest X-ray, *CT* computed tomography, *COVID*-*19* Coronavirus disease 2019, *HRCT* high-resolution computed tomography, *RT*-*PCR* reverse-transcription–polymerase chain reaction

Two hundred eighty-six COVID-19 patients who presented to our hospital between March 15, 2020 and September 1, 2020 were consecutively collected. The inclusion criteria were as follows: (1) positive result of RT-PCR testing, (2) having CXR examination with a preliminary diagnosis of pneumonia, and (3) having CT examination additional to CXR. The exclusion criteria were as follows: (1) negative chest CT imaging, (2) long interval between CT and CXR imaging (more than 24 h), (3) CT image acquisition other than the high-resolution protocol, (4) low-quality radiologic images that prevent scoring, and (5) being under the age of 18. Of 286 patients, 63 with negative chest CT findings, 10 with CT angiographic images, 8 with low-quality radiologic images, 90 with a time interval of more than 24 h between CT and CXR imaging, and 2 under the age of 18 were excluded. Finally, 113 patients (59 male, 54 female, mean age = 55.28 ± 15.01), who met these criteria were included in the study. The patient selection process is summarized in Fig. 1.

### Image acquisition and interpretation

Computed tomography images were acquired with a 160-slice-CT scanner (Aquilion Prime, Toshiba Medical Systems, Tokyo, Japan) using high-resolution CT protocol. The scanning parameters were 120 kVp, 80 × 0.5 mm collimation, automated dose reduction, reconstruction with 0.5 mm slice thickness. The axial images were taken at deep inspiration during a single breath-hold and included the body part from the thoracic inlet to the diaphragm. Chest X-ray images were obtained in the upright position (posterior-anterior view) or in the supine position (anterior-posterior view).

Two couples of radiologists evaluated the chest CT and CXR images separately. Two radiologists with 6 and 16 years of experience in thoracic imaging evaluated CT images independently. The other two radiologists with 15 and 25 years of experience in thoracic imaging, who were blinded to patients’ clinical situation and CT imaging results, evaluated CXR images independently. Final decisions were reached by consensus in both groups.

### Scoring system

**Fig. 2:**
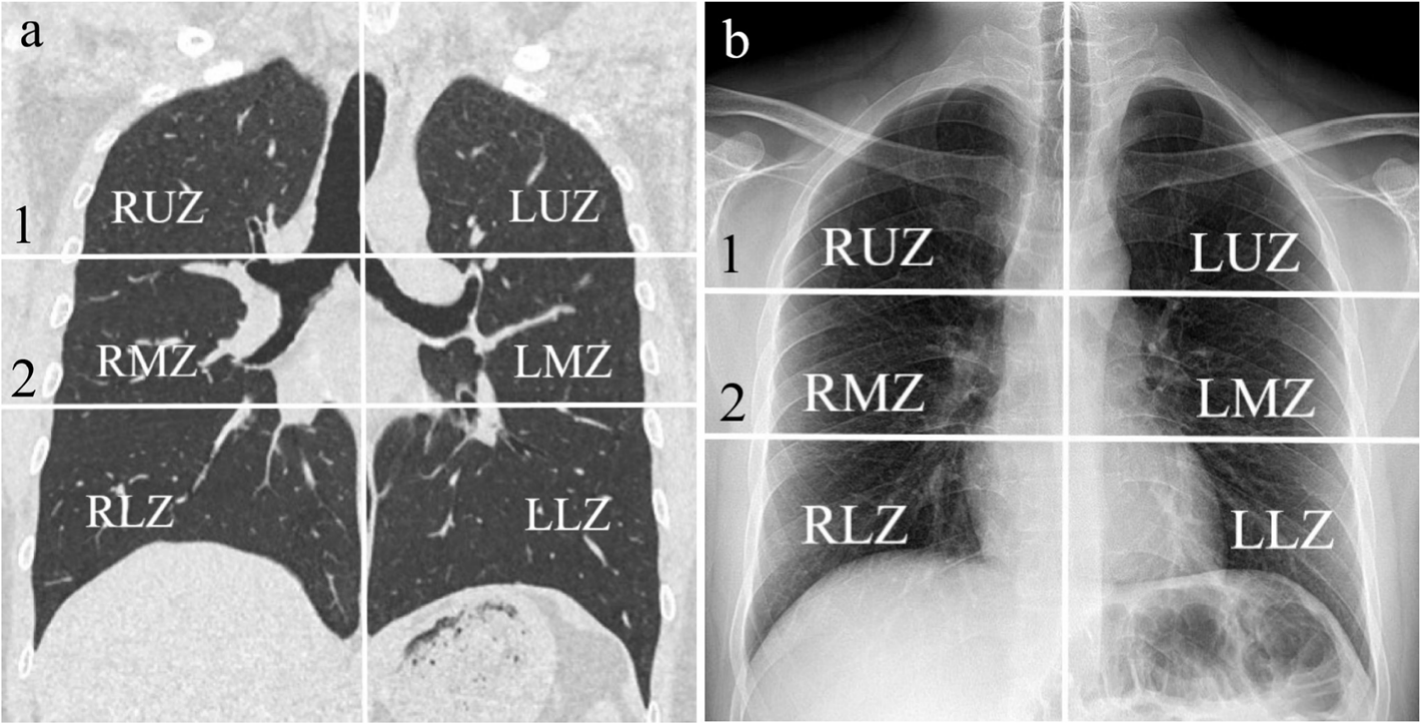
**a**, **b** Lung zones. Two lines, one drawn at the carina level (1) and the other drawn at the level of the inferior wall of the right inferior pulmonary vein (2) divide the lung into 6 zones on both coronal reformatted CT (**a**) and CXR (**b**) images. *LLZ* left lower zone, *LMZ* left middle zone, *LUZ* left upper zone, *RLZ* right lower zone, *RMZ* right middle zone, *RUZ* right upper zone

In literature, there are several scoring systems established for COVID-19 pneumonia focusing on either CT or CXR [7, 10, 15–19]. With a different approach, we aimed to design a combined scoring system based on criteria matching each other in these two imaging modalities. Our CXR scoring method shows similarities with the Brixia scoring system defined by Borghesi et al [15]. However, some modifications have been done to make it concordant with the CT score considering the well-known CT features of COVID-19 pneumonia such as GGO, crazy paving pattern, and consolidation. We divided the lung into six zones on both chest CT and CXR images: right lower zone, right middle zone, right upper zone, left lower zone, left middle zone, and left upper zone. We used the same division criteria for the two modalities to ensure optimal compatibility. Upper zones were defined as the area above the carina. Lower zones were defined as the area below the level of the inferior wall of the right inferior pulmonary vein. Middle zones were determined as the lung parenchyma between the upper and lower zones (Fig. 2). Then, per each zone, we used a grading system based on the density and infiltration patterns of the lesions, as the most favorable way to adapt the scoring system to these different modalities. Ground glass opacities on the CT images, corresponding hazy densities on the CXRs, were ‘grade 1’ lesions equal to ‘1 point.’ Crazy paving pattern and reticular densities on the CT images were ‘grade 2’ lesions equal to ‘2 points.’ Reticular pattern was the equivalent of this grade for CXRs. Finally, consolidation was a ‘grade 3’ lesion equals to ‘3 points.’ In the case of the presence of mixed type lesions in the same zone, the lesion with the highest grade was included in the scoring system. Using these criteria, we obtained zonal scores ranging from 0 to 3 and calculated total CT score (CTS) and X-ray score (XRS), ranging from 0 to 18 per patient. Table 1 demonstrates the grading system mentioned above.

**Table 1:** Grading system based on the density and morphology of the lesions

CXR finding	CT finding	Grade	Score
None	None	0	0
Hazy density	Ground glass opacity	1	1
Reticular density	Crazy paving pattern, reticular density	2	2
Consolidation	Consolidation	3	3

In the case of the presence of mixed type lesions in the same zone, the lesion with the highest grade was included in the scoring system. *CXR* chest X-ray, *CT* computed tomography

### Statistical analysis

All statistical analyses were performed using IBM SPSS version 25.0. Kolmogorov-Smirnov and Shapiro-Wilk tests were used for the assessment of the normality of numerical variables. Descriptive statistics included means and standard deviations (SDs) for normally distributed variables. Median, minimum, and maximum values were calculated for non-normal distributed variables. The median values were compared using the Mann-Whitney *U* test and mean values were compared using the two-sample *t* test. Spearman correlation test was performed for correlation analyses. The intraclass correlation coefficient (ICC) was used to assess interobserver reliability. ICC values between 0.75 and 1.00 suggest an excellent correlation, between 0.60 and 0.74 good correlation, and < 0.4 poor correlation. Receiver operator characteristics curve (ROC) analysis was used for the determination of the threshold values. The confidence interval (CI) was regarded as 95% and statistical significance was defined as a p-value less than 0.05.

## Results

### CT and CXR findings

The current study included 113 COVID-19 patients (59 male, 54 female, mean age = 55.28 ± 15.01). Since it was one of our inclusion criteria, all patients had lung infiltration suggesting pneumonia on their CT images. Of these 113 CT scans, 110 (97.3%) showed lower zone involvement. Left upper zone was the least affected region (69.0%). Most of the patients had bilateral lung infiltration on CT scans (92.9%). Seventy-nine patients (69.9%) also had findings on their CXR supporting the diagnosis of pneumonia. Bilateral lung involvement was observed in 58 of 79 CXRs (73.4%). Lower zones were the most affected areas (96.2%). Hazy densities were the most common pattern observed in the affected zones (45.6%). On CXRs, 250 of 678 zones showed signs of infiltration (36.9%). This number was 561 (82.7%) on CT scans. At 334 of these 561 zones, GGO, which was described as a grade 1 lesion in our grading system, was the only pattern. On CXRs, 262 of these 334 zones (78.4%) were clear. This ratio tended to increase from the lower zones (right lower zone = 55.9%, left lower zone = 66.0%) to the upper zones (right upper zone = 93.3%, left upper zone = 96.7%). At 227 zones, grade 2 and 3 lesions (crazy paving pattern, reticular opacities, consolidation) were observed on CT images. Among them, 160 zones (70.4%) showed parenchymal density changes consistent with pneumonia on CXRs. Table 2 shows the zonal distribution of lesions integrated into the grading system.

**Table 2:** Distribution of the lesions integrated into the grading system, number of involved zones (*n*), and percentages (%) (*n* = 113, total zone = 678)

	Grade 1		Grade 2		Grade 3		Total	
	CXR	CT	CXR	CT	CXR	CT	CXR	CT
RLZ	26.5% (30)	52.2% (59)	20.4% (23)	17.7% (20)	14.2% (16)	23.9% (27)	61.0% (69)	93.8% (106)
RMZ	23.9% (27)	45.1% (51)	12.4% (14)	24.8% (28)	10.6% (12)	20.4% (23)	46.9% (53)	90.3% (102)
RUZ	8.0% (9)	53.1% (60)	5.3% (6)	15.9% (18)	5.3% (6)	7.1% (8)	18.6% (21)	76.1% (86)
LLZ	24.8% (28)	46.9% (53)	15.9% (18)	20.4% (23)	10.6% (12)	17.7% (20)	51.3% (58)	85.0% (96)
LMZ	13.3% (15)	45.1% (51)	8.0% (9)	23.0% (26)	9.7% (11)	14.2% (16)	31.0% (35)	82.3% (93)
LUZ	4.4% (5)	53.1% (60)	2.7% (3)	10.6% (12)	5.3% (6)	5.3% (6)	12.4% (14)	69.0% (78)
Total	16.8% (114)	49.2% (334)	11.6% (73)	18.7% (127)	9.2% (63)	14.7% (100)	36.9% (250)	82.7% (561)

*CXR* chest X-ray, *CT* computed tomography, *LLZ* left lower zone, *LMZ* left middle zone, *LUZ* left upper zone, *RLZ* right lower zone, *RMZ* right middle zone, *RUZ* right upper zone

### Evaluation of the scores

**Fig. 3:**
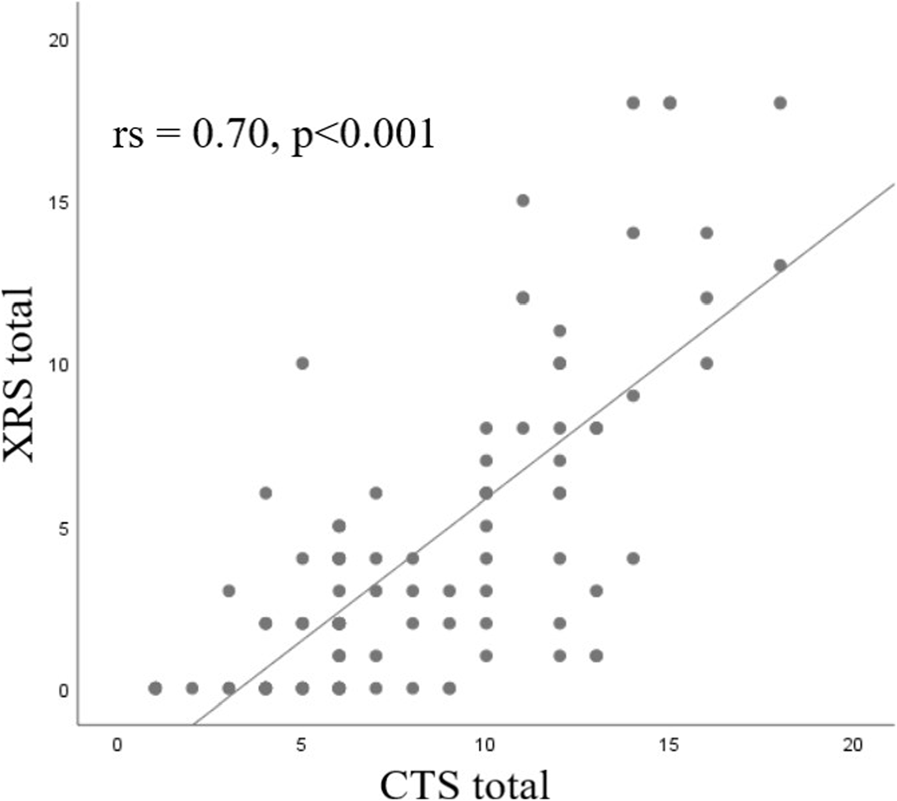
Scatter plot graph of the correlation between total scores. The graph shows the high positive correlation between total CTSs and XRSs (rs = 0.70, *p* < 0.001). *CTS* computed tomography score; *XRS* X-ray score

**Fig. 4:**
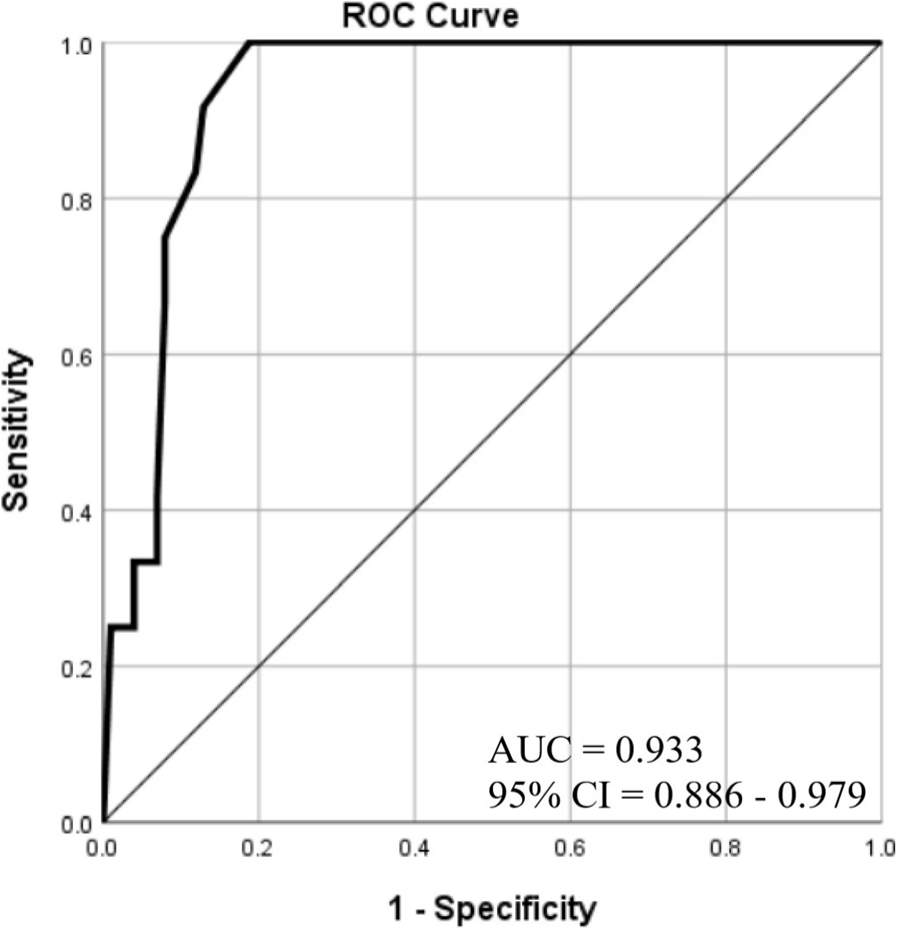
ROC curve of total XRS to predict intensive care necessity. The cut-off value of XRS in distinguishing patients who needed intensive care in the follow-up period was 6 (AUC = 0.933, 95% CI = 0.886 - 0.979). This cut-off value had 100% sensitivity, 81% specificity. AUC, Area under curve; CI, Confidence interval; ROC, Receiver operator characteristics

**Fig. 5:**
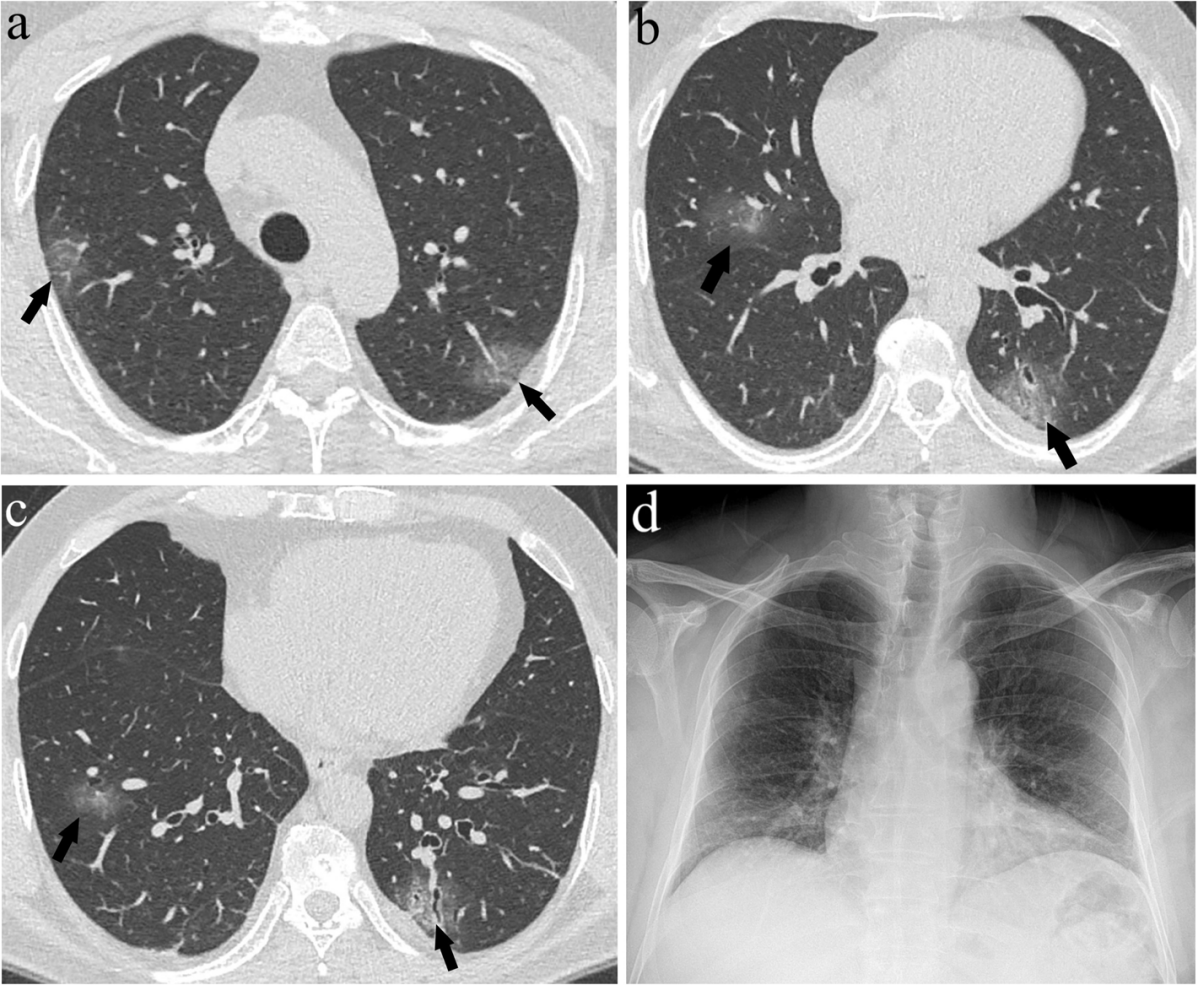
**a**–**d** A 56-year-old male presenting with dry cough and fever for 3 days. Axial CT images at the level of the upper (**a**), middle (**b**), and lower (**c**) lung zones demonstrate ground glass opacities representing grade 1 lesions (arrows) (CTS = 6). There were no signs of pneumonia on the CXR image (**d**)

**Fig. 6:**
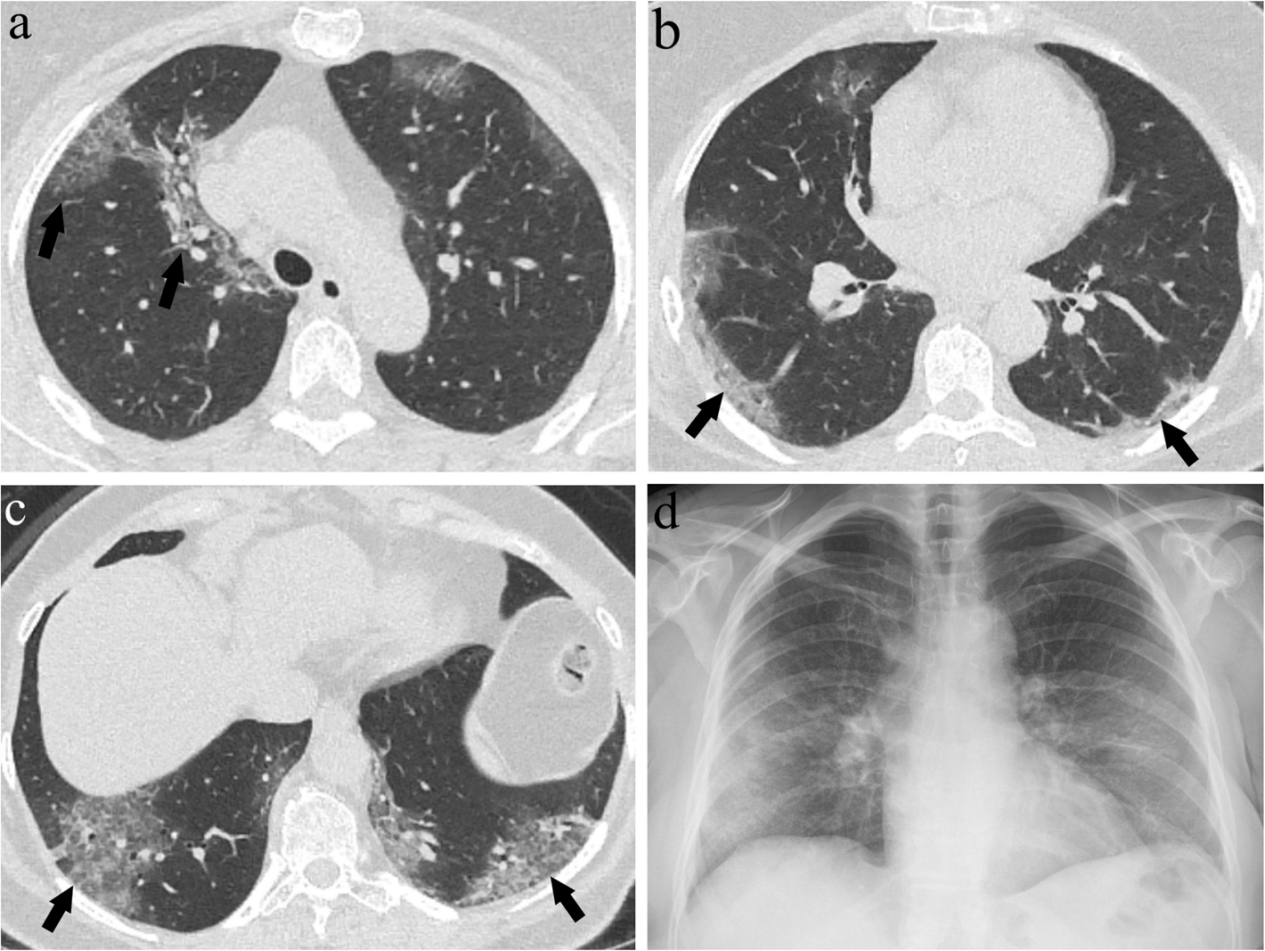
**a**–**d** A 63-year-old female presenting with mild symptoms; dry cough and fatigue. Axial CT images at the level of the upper (**a**), middle (**b**), and lower (**c**) lung zones show lesions predominantly distributed in peripheral areas. Additional to ground glass opacities, the crazy paving pattern and consolidations representing grade 2 and 3 lesions (arrows) were present. CXR image (**d**) shows peripheral hazy opacities and reticular pattern. Despite mild clinical findings, this patient had a CTS of 12 and XRS of 7 and needed monitoring in the intensive care unit during the follow-up period

**Fig. 7:**
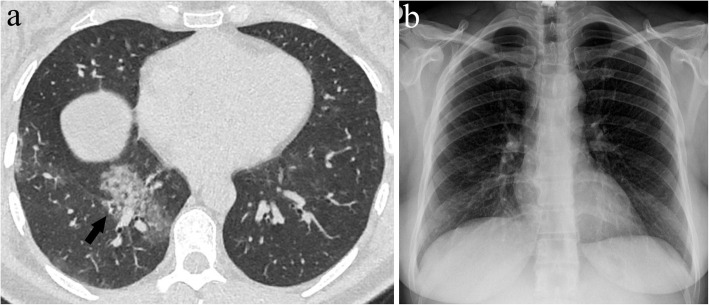
**a**, **b** A 43-year-old female presenting with fever and cough. Axial CT image at the level of lower lung zones (**a**) shows an area of consolidation in the right lower lobe below the level of the dome of the diaphragm (arrow). There is no observable opacity on the CXR image (**b**), revealing the limitation of CXR in demonstrating pathologies located in such blind spots

The sum of total CTSs of 113 patients (888, range 1 to 18, median = 6) was higher than that of XRSs (449, range 0 to 18, median = 2). We found a high positive correlation between total CTSs and XRSs (rs = 0.70, *p* < 0.001) (Fig. 3). Also, there were low to moderate positive correlations between CTSs and XRSs of each zones. The sum of CTSs of right lower zone (180) was the highest and left upper zone (102) was the lowest. Similar results were obtained in the CXR scoring. Sum of XRSs of right lower zone (124) was the highest and left upper zone (29) was the lowest (Table 3). Patients with a normal CXR (*n* = 34) had significantly lower CTS than patients with a CXR showing signs of pneumonia (*n* = 79) (*p* < 0.001). The CTS cut-off value of 7 had 62% sensitivity and 88% specificity for the differentiation of positive from negative CXRs (AUC = 0.863, 95% CI = 0.795–0.930). Among our study patients, 12 of them were referred to the ICU during the follow-up period. Total CTSs (median = 13.5) and total XRSs (median = 10) were high and close to each other in these patients. Computed tomography scores of ICU patients were significantly higher than those of non-ICU patients (*p* < 0.001). Similarly, XRSs of ICU patients were significantly higher than those of non-ICU patients (*p* < 0.001) (Table 4). The cut-off value of XRS to distinguish ICU patients from non-ICU patients was 6 (AUC = 0.933, 95% CI = 0.886–0.979). This cut-off value had 100% sensitivity, 81% specificity (Fig. 4). Representative cases are shown in Figs. 5, 6, and 7. The interobserver reliability of X-ray scoring was excellent with an ICC of 0.95 (95% CI 0.93–0.97). Similarly, the test results showed an excellent correlation for CT scoring with an ICC of 0.96 (95 % CI 0.94–0.97).

**Table 3:** Zonal and total CTSs and XRSs; sum of values, medians, correlation coefficients (rs) (*n* = 113)

Lung zone	RLZ	RMZ	RUZ	LLZ	LMZ	LUZ	Total	Median	*p* value
CTS	180	176	120	159	151	102	888	6	–
XRS	124	91	39	100	66	29	449	2	–
Correlations (rs)*	0.56	0.47	0.44	0.45	0.47	0.46	0.70	–	*p* < 0.001

*Spearman correlation coefficient (rs); 0.3–0.5 low, 0.5–0.7 moderate, 0.7–0.9 high correlation. *p* < 0.05 shows statistical significance. *CTS* computed tomography score, *LLZ* left lower zone, *LMZ* left middle zone, *LUZ* left upper zone, *RLZ* right lower zone, *RMZ* right middle zone, *RUZ* right upper zone, *XRS* X-ray score

**Table 4:** Comparison of patient groups based on CXR positivity and intensive care necessity (*n* = 113)

RT-PCR positive patients (*n* = 113)	CXR (+) (*n* = 79)	CXR (−) (*n* = 34)	*p* value*	ICU (−) (*n* = 101)	ICU (+) (*n* = 12)	*p* value*
Gender	46 M, 33 F	13 M, 21 F	–	53 M, 48 F	6 M, 6 F	–
Age (mean ± SD)	59.51 ± 14.04	45.47 ± 12.54	p < 0.001	53.61 ± 14.36	69.33 ± 13.31	*p* < 0.001
CTS (median)	10	4.5	p < 0.001	6	13.5	*p* < 0.001
XRS (median)	4	–	–	2	10	*p* < 0.001

*p values calculated by two-sample *t* test and Mann-Whitney *U* test, *p* < 0.05 shows statistical significance. *CXR* chest X-ray, *CTS* computed tomography score, *ICU* intensive care unit, *RT*-*PCR* reverse transcription–polymerase chain reaction, *XRS* X-ray score

## Discussion

The aim of our study including 113 confirmed COVID-19 patients was to assess the role of CXR in the diagnosis of pneumonia as well as its value in making the triage of patients at their initial presentation to the hospital. The cut-off value that we have found using our scoring system showed high sensitivity and specificity in distinguishing patients who needed intensive care during the follow-up period, suggesting that a baseline CXR taken after admission can be a valuable tool in the management of the disease. The correlations between CT and CXR scores showed the consistency of this scoring system in itself and also indicated that both modalities provide similar insights into the extensity of the parenchymal involvement.

Several previous studies showed the lower sensitivity of CXR and the higher sensitivity of chest CT when compared with RT-PCR [11, 12, 17]. Wong et al. [11], in their study including 64 RT-PCR positive patients, reported the lower sensitivity of CXR (69%) than RT-PCR (91%). Kim et al. [20] reported a lower positivity rate of 46.4% when compared with our results (69.9%). Differently, our findings also included the zone-based evaluation. Among the lung zones where the only infiltration pattern was pure GGO on CT images, the ratio of detecting pathology on CXRs was much lower (21.6%). However, this ratio increased to 70.4% when the lesions observed on CT images were consolidation and/or crazy paving pattern. In addition, the high specificity and relatively low sensitivity of CTS cut-off value in distinguishing positive and negative CXRs revealed the limitation of CXR in detecting the lesions, particularly those at lower densities. These findings suggest that GGOs, the most common features of COVID-19 pneumonia especially at the initial stage, are the cause of the low sensitivity of CXRs. Previous publications showed that bilateral, multifocal, and peripheral distribution of the lesions is a common pattern of the disease [17, 19, 21]. This distribution pattern of the disease may increase the detectability of the lesions on CXR images, particularly at the progressive stages when the density of the lesions increases and can be more easily detectable on CXRs.

Patients in the ICU, who are expected to be at the progressive stage, can be monitored with CXR confidently, given its advantages. The European Society of Radiology and the European Society of Thoracic Imaging advised that it is appropriate to use CXRs for follow-up of the patients monitoring in the ICU, rather than being used as a primary care imaging technique [22]. Jacobi et. al. [23] suggest that lung infiltration patterns can be identified on CXR, and grading the severity of COVID-19 based on lung involvement is of importance to relay to the clinicians. In addition, considering the high sensitivity and specificity of the XRS cut-off value to distinguish ICU patients from non-ICU patients, we suggest that the initial CXR taken after admission to the hospital can predict the patients to be monitored in the ICU. We also think that it will be useful to have a baseline CXR so that the comparison with follow-up radiographs can be performed in full compliance. Borghesi et al. [15] mentioned the importance of CXR imaging as a diagnostic tool that can be useful for monitoring patients ‘day after day’ for the rapid progression of lung abnormalities. However, it should not be forgotten that, even though not as much as CT, CXR is an imaging method that causes radiation exposure. Chest X-ray may be useful for assessing disease progression in hospitalized patients, however, daily routine examinations are not indicated in stable intubated patients due to radiation concern [24].

Even though GGO, crazy paving pattern, consolidation are the common patterns of the disease, there are additional findings shown in the literature to be observed on chest CT images, which were not included in our scoring system such as; halo sign, reverse halo sign, vascular enlargement, air bubble sign, bronchiectasis, subpleural line, and pleural thickening [25, 26]. These findings may not be obviously visible on CXR images. Also, extra-parenchymal findings showing higher incidences among severe and critical patients such as mediastinal lymphadenopathy, pleural effusion, and pericardial effusion can be observed more easily on CT images [18]. Apical, hilar, retrocardiac regions, and the region below the dome of the diaphragm are blind spots. Lesions located in these areas may be overlooked on the CXRs [27]. Particularly, ‘the region below the dome of the diaphragm’ is of importance, considering the most common distribution pattern of the lesions in COVID-19 pneumonia [28]. Besides showing GGOs clearly, these additional advantages of CT over CXR increase the tendency to this modality. However, high radiation exposure is a fact that should be considered. Concerning this matter, the Fleischner Society released a multinational consensus statement. This statement has focused exclusively on the use of not only CT but also CXR. They mentioned that radiologic imaging is indicated in a limited patient group; COVID-19 patients with worsening respiratory symptoms and moderate-severe clinical features, COVID-19 suspected cases with moderate-severe clinical features, and/or a high pre-test probability of disease in case of unavailable RT-PCR testing [24].

This study had several limitations. Firstly, the scoring system is based on visual interpretation and observer-dependent. It needs validation with larger patient groups. Even though changes in the density of the lesions are indicative of progression, the extension of the lesions is also a sign of progression. However, to adapt it to both chest CT and CXR, we preferred to design the scoring system based on density changes only. Multizonal involvement was the only indicator of extensity. Secondly, the time interval between chest CT and CXR imaging was up to 24 h. A rapid progression that could be seen in this time period was underestimated; however, this situation was within the bounds of possibility. Thirdly, the number of patients monitored in the ICU was limited. A larger patient group could further increase the statistical power of the study.

## Conclusions

In conclusion, ground glass opacities, the most common CT features of COVID-19 pneumonia especially at the early stage, are the cause of low sensitivity of CXRs. However, at the progressive stages, in parallel with the increase in lesion density, lesions can be detected more easily on CXRs. We suggest that a baseline CXR taken after admission to the hospital can predict the patients to be monitored in the intensive care units. Besides, it will be beneficial to have a baseline CXR in terms of making a comparison with follow-up radiographs in full compliance.

## Data Availability

All data generated or analyzed during this study are included in this published article.

## Abbreviations

COVID-19: Coronavirus disease 2019
RT-PCR: Reverse-transcription–polymerase-chain-reaction
CT: Computed tomography
GGO: Ground glass opacity
CXR: Chest X-ray
CTS: Computed tomography score
XRS: X-ray score
RLZ: Right lower zone
RMZ: Right middle zone
RUZ: Right upper zone
LLZ: Left lower zone
LMZ: Left middle zone
LUZ: Left upper zone
ICU: Intensive care unit
ICC: Intraclass correlation coefficient
ROC: Receiver operator characteristics
CI: Confidence interval
AUC: Area under curve
